# One of the Results of the Administration of Kairin

**Published:** 1884-12

**Authors:** R. J. Nunn

**Affiliations:** Savannah, Ga.


### ONE OF THE RESULTS OF THE ADMINISTRATION OF KAIRIN.

BY R. J. NUNN, M. D., SAVANNAH, GA.

A child of five years of age was being treated for malarial fever of the continued type; pulse ranging about 125, temperature 105 degrees F, and over. Five grains of kairin were given every two hours until thirty grains were taken. This was followed by a fall of temperature to 101 degrees F, but the urine passed was blue, commencing about twelve hours after the administration of the kairin, continuing about twenty-four hours, and then gradually resuming the normal appearance; the reaction of the urine was acid sp. g. 1,020, no albumen. The addition of nitric acid turned the blue color to a deep red. After standing forty-eight hours in a corked bottle the blue urine gradually assumed a normal appearance, the change commencing at the bottom of the bottle and gradually extending to the top.

The reduction of temperature was of short duration, very much shorter than I have been accustomed to see after the administration of kairin.

This result was so unexpected that I was anxious to assure myself that it was dependent upon the kairin as a cause, and as a test repeated the kairin after a few days and its administration was followed by a like result.

Although I have used kairin very extensively with patients of all ages, I have never in any other case observed results similar to those just described.

The facilities for chemical analysis at my command were much too meagre for me to attempt an investigation looking to the discovery of the nature of the substance which gave to the urine in question the abnormal appearance described. I must therefore content myself with recording the fact, in the hope that attention being directed to the urine changes resulting from the administration of kairin, others more favorably situated than myself may have an opportunity to thoroughly investigate the subject. I would suggest, however, that a clue to the solution of the problem may be found in the parallel action of carbolic acid.

